# Deferasirox and Ciprofloxacin: Potential Ternary Complex Formation With Ferric Iron, Pharmacodynamic, and Pharmacokinetic Interactions

**DOI:** 10.1155/tswj/9309491

**Published:** 2024-11-29

**Authors:** Imad I. Hamdan, Ruba T. Tarawneh, Firas Awwadi, Duaa Al-Qattan, Ahmad I. Hamdan, Fatma Afifi

**Affiliations:** ^1^Department of Pharmaceutical Sciences, The University of Jordan, Amman, Jordan; ^2^Department of Chemistry, The University of Jordan, Amman, Jordan; ^3^School of Medicine, The University of Jordan, Amman, Jordan

**Keywords:** chelate complexes, ciprofloxacin, deferasirox, drug interaction, iron overload

## Abstract

The main aim of this work was to assess the potential formation of ternary chelate complexes, involving deferasirox (DFX), ciprofloxacin (CP), and ferric iron. The coadministration of CP along with DFX might modulate its efficacy, so it is important that it be investigated. A ternary complex involving DFX, CP, and iron (DFX-CP-Fe) was prepared and characterized. Theoretical chemistry calculations were performed to measure the equilibrium constants of complexes. Two groups of rats were exposed to DFX or DFX with CP. The level of DFX in plasma was measured, and histological assessments of relevant organs were made. Levels of iron in selected tissues were measured. The formation of ternary complexes was confirmed. Two ternary complexes are thermodynamically favored. The first one of ratio (DFX:CP:Fe) was shown to be favorable with an equilibrium constant of 2 × 10^7^. The second one with ratio (DFX:CP:2Fe) is more thermodynamically favored with an equilibrium constant of 2.0 × 10^60^. Rats treated with a combination of DFX and CP exhibited lower levels of iron in dried red blood cells in comparison to those treated with DFX alone (*p* value = 0.012). They also exhibited lower levels of DFX in plasma. Histological assessments of the relevant tissues showed a clear difference in the level of deposited iron in the spleen. In conclusion, ternary complexes are formed, and some with exceptionally high constants. The obtained data indicate a potentially favorable role of CP because while it resulted in a decrease in the level of DFX, it pharmacodynamically produced more effect.

## 1. Introduction

**Figure 1:**
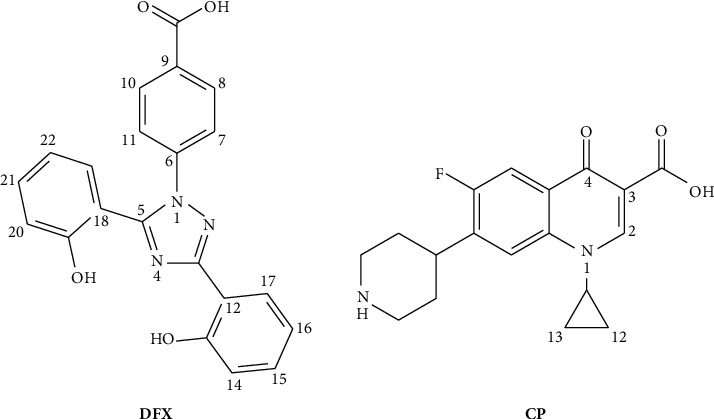
Structures of DFX and CP.

Deferasirox (DFX, Figure 1) is an iron-chelating agent used to treat iron overload in patients suffering from thalassemia and other chronic conditions when patients were subjected to multiple blood transfusions [1, 2]. In the absence of adequate chelation therapy for iron elimination, excess iron from frequent transfusions can accumulate in numerous organs, causing major damage and eventually death [3–6].

The pharmacological efficacy of DFX has been demonstrated in iron-overloaded animals [7, 8]. Previous studies have shown that DFX binds Fe^3+^ with a stoichiometry of two ligand molecules to one atom of the metal so that the coordinate bonds are formed via the two phenolic groups and the isolated nitrogen in each of the two coordinated molecules in the complex [9, 10]. The practically measured formation constants of DFX to Fe^3+^ were exceptionally high, mounting to 10^37^-10^38^ although very low affinity of DFX to Fe^2+^ has been reported [11, 12].

DFX–iron complex possesses different physicochemical properties than the free drug, including higher molecular weight and lower water solubility [13]. Therefore, the iron containing complex is mainly eliminated via hepatic bile [14, 15]. DFX has low water solubility and variable bioavailability when taken together with food [16]. Combined use of DFX with deferoxamine, another clinically used iron chelator, has been studied in terms of safety and efficacy. The combination was well tolerated without any additive effect of iron removal capacity [17, 18]. Unfortunately, DFX has been accompanied with a high fatality rate due to its dangerous side effects on the liver, kidneys, and the digestive system [19, 20]. Moreover, it has been reported that it is not efficient in removing iron from the heart which may eventually lead to increased morbidity [21].

Ciprofloxacin (CP) is a fluoroquinolone antibiotic (Figure 1) used to treat various bacterial infections [22]. Due to the presence of electron donating groups in its structure, it has the ability to form complexes with metal ions including iron. CP–metal complexes can have different properties and may affect the pharmacological activity of CP [23–26]. Studies have shown that CP binds to iron strongly enough to enable removal of excess iron from the body by excreting the formed complex through urine [24, 25].

Since both DFX and CP have the ability to bind to iron, we were interested in investigating the possibility of ternary complex formation involving iron, DFX, and CP inside the body in the cases of potential concomitant use for the treatment of iron overload. This in turn might lead to an improved or deteriorated efficacy of iron removal. If a synergistic effect was demonstrated, then it could be recommended to use the combination of both drugs for iron removal in some cases. The present work describes synthesizing and characterizing a ternary DFX-CP-Fe complex. Additionally, the effect of concomitant use of CP with DFX in an iron-overloaded rat model was investigated and discussed.

## 2. Materials and Methods

DFX and CP hydrochloride were gifts from Al Hikma Pharmaceutical Company (Amman-Jordan) and Jordan Pharmaceutical Manufacturer (JPM, Naur, Jordan), respectively. HPLC grade solvents were obtained from Fisher Chemicals (Loughborough, UK) while carbonyl iron and other used buffer materials and reagents were purchased from Sigma-Aldrich (USA).

### 2.1. Preparation of the Complexes

For preparation of the DFX-Fe^3+^ complex, 0.62 g of DFX was added to 50 mL of 30% MeOH in H_2_O. The mixture was maintained at 50°C with continuous mixing while the pH was adjusted to 8.2 using trimethylamine. The solid dissolved completely in less than 2 minutes. In a separate flask, 0.27 g of FeCl_3_, equimolar to the amount of DFX, was completely dissolved in 40 mL of 30% MeOH by sonication before they were added to the drug solution while mixing with a magnetic stirrer. The solution immediately turned dark violet in color. The pH of the solution was adjusted to 7.5 before it was left to stand at room temperature (RT) for 2 h. The formed dark precipitate, which is the potential DFX-Fe^3+^ complex, was collected by filtration and left to dry over calcium chloride for > 48 h. For the preparation of the potential ternary complex “DFX-CP-Fe^3+^,” the above described procedure was repeated with the some modifications. Ferric chloride (0.27 g) was added to 50 mL of 0.61 g CP and dissolved in 30% MeOH. The solution, which turned immediately reddish in color, was slowly added to the solution of DFX by stirring using a magnetic stirrer. The pH of the solution was adjusted to 7.5, and a precipitate started to form. The brown precipitate, assumed as the ternary complex, was collected by filtration and dried over CaCl_2_.

### 2.2. FTIR, ^1^H-NMR, and DSC

In addition to measuring the melting points of the obtained products and using the standard capillary melting point apparatus, differential scanning calorimeter (DSC) thermograms were obtained using a Mettler–Toledo DSC823e calorimeter (Mettler Toledo, Switzerland) configured to a Mettler Star software system. Thermograms were obtained in the range of 0–300°C with an increasing rate of 10°C/min. FTIR spectra were recorded using a Shimadzu FTIR spectrophotometer (Japan) using the KBr disk method. ^1^H-NMR spectra were obtained using a Perkin Elmer 500 MHz NMR spectrophotometer (USA).

### 2.3. Determination of Molar Ratio of Association

The molar ratio of association between iron and either of the drugs was determined by completely dissolving 2.7 mg of the metal complex products in 10 mL of a mixture of butanol, DMSO, and DMF (3:5:2). Solutions were then diluted twice in DMSO and five times in mobile phase before being injected into HPLC. The developed HPLC method was validated for this purpose and for the determination of solubility and partition coefficients (PCs). The optimum conditions developed comprised a phenyl column (4.6 mm ID; 150 mm·L; Thermo Scientific Hypersil) with a mobile phase consisting of 34% acetonitrile in a 50 mM phosphate buffer. The mobile phase mixture contained 0.032% citric acid, 0.02% EDTA, 0.032% hydroxylamine HCl, and 0.07% trimethylamine, with the overall pH adjusted to 3.7. The mobile phase was delivered isocratically at a flow rate of 1.2 mL/min, and chromatograms monitored at 290 nm.

The method was validated for selectivity, precision, accuracy, and linearity with satisfactory results. Selectivity was established by demonstrating perfect resolution between CP and DFX, and neither the buffer components nor any of the drugs coeluted at the same time (chromatograms are shown in Figure 1S, Supporting Information, Representative HPLC chromatograms for the determination of DFX in solubility studies). Linearity was satisfactory with *R*^2^ > 0.99 in the range 1–60 μg/mL for DFX. Typical calibration equations for DFX were *y* = 29*x* + 0.001.

For precision, five quality control samples of each drug were prepared at low, intermediate, and high concentration levels. The obtained RSD values were always < 1.8 and thus concluded satisfactory. The lowest limit of quantification (LOQ) was determined for DFX based on standard solutions which provided reasonable precision and accuracy when concentration was back-calculated with the adopted linear equation. The adopted LOQ value for DFX was 1.0 μg/mL. Relevant calibration equations were employed to determine the average concentrations of DFX (μg/mL) in the samples. Thus, the number of moles of the DFX and eventually the molar ratio between the components could be estimated.

### 2.4. Spectroscopic Titration of the Drugs With Ferric Iron

All UV measurements were made using a Cary UV spectrophotometer. A stock solution of DFX (8.0 mM) was prepared in 20% MeOH in distilled H_2_O, and the pH was adjusted to 8.2 using a 0.2 M Tris base solution. A stock solution of FeCl_3_ was prepared in distilled H_2_O at a concentration of 18.5 mM. A constant volume of the stock DFX solution (1.5 mL) was transferred into 8 sample tubes. Before completing the volumes of all tubes to 40 mL, increasing volumes of the stock FeCl_3_ solution were added (0–4.0 mL) so that the ratios between iron and DFX (*r*) increased within the range 0- ∼8. The pH was adjusted to the required value (7.4, 5.2, or 2.5), and the volumes in all tubes were made up to 40 mL. Accordingly, the concentration of DFX in each tube was fixed at 0.3 mM. For the titration of CP alone, a second set of samples was prepared in a similar manner but using 0.6 mL of a stock solution of CP in distilled H_2_O (20 mM) which would be equimolar to the analogous DFX solutions.

A third set of samples was also prepared similarly but containing a mixture of both drugs at equimolar amounts. A fourth group of titration solutions was prepared to contain only the equivalent amount of iron that existed in the corresponding sample tubes. This latter group served as a blank to examine how the increasing concentrations of iron on its own would change the absorbance profile. Because of the appearance of turbidity in some samples, all samples were diluted the same way before measurements; that is, 4 mL of the sample solution (after vortex mixing) was added to 1 mL of triethylene glycol. Absorption spectra were recorded in the range of 240–800 nm, and absorption was recorded for each sample at the selected wavelengths. For each set of samples for titration, the experiment was repeated three times. The reproducibility was quite high (RSD < 2.2%). Molar ratio plots were obtained for each set of samples by plotting the obtained average absorbance value against the molar ratio of added iron to the drug.

### 2.5. Determination of Solubility and PCs

Excess solid of each compound to be tested was added to 6 mL of either phosphate buffer (pH 6.8), acetate buffer (pH 4.5), or 0.1 M HCl and left on a shaker water bath (37°C) for 24 h. One mL was centrifuged for 10 min, and 100 μL of the supernatant was properly diluted with the mobile phase (1:4) before being injected onto HPLC. For determination of octanol/H_2_O PC, 0.6 mL of the supernatant solution was transferred to a new tube containing 2 mL of octanol. Tubes containing the two layers of octanol and aqueous phase were kept on shaker water bath (37°C) for 24 h. Aliquots of aqueous layer were properly diluted with the mobile phase before being injected onto HPLC. The concentration of the drug in octanol layer was calculated by difference [27].

### 2.6. Theoretical Chemistry Calculations

The thermodynamics of the two reactions

(1) $$\begin{array}{c} {\text{FeCl}}_{3}+\text{CP}+\text{DFX}+H_{2}\;O⟶\left(\text{DFX};\text{ CP};\text{ Fe}\right)+3\text{HCl} \end{array}$$

(2) $$\begin{array}{c} 2{\text{FeCl}}_{3}+\text{CP}+\text{DFX}+3\;H_{2}\;O+{\text{OH}}^{-}⟶\left(\text{DFX};\text{ CP};\;2\text{Fe}\right)+4\text{ HCl}+{\text{Cl}}^{-} \end{array}$$

were studied theoretically using the G09 package [28]. The structure of the reactants and products was optimized at the DFT/B3YP level. Cc-pVDZ basis sets were assigned for C, H, O, and F and LANL2DZ for Fe^3+^. The thermodynamic parameters were calculated as instructed in the manuscript published on the Gaussian website [29].

### 2.7. Animal Studies

Ethical approval of the study protocol was obtained from the Institutional Review Board at the University of Jordan (Approval No. 47–2022) on 17th May 2022 (document No. 19/2022/289). Throughout the study, all animals were housed, fed, and treated in accordance with The University of Jordan ethical guidelines for animal protection. The animal house of the University of Jordan, which is equipped with proper ventilation and temperature control (25 ± 4°C) systems, was utilized for the study. Each animal was placed in a separate standard cage. Sixteen male Wistar rats (6 weeks old, average weight 220 ± 10 g) were subjected to an iron-enriched diet containing 2.5% carbonyl iron according to Ackerman et al. [30] for 6 weeks. Another group of eight rats (control group) was subjected to an ordinary pellet diet for the same period. The 16 animals with iron fortified diets were then separated into two groups of eight: the DFX group which received DFX alone and the DFX-CP group which received DFX plus CP, for 6 days. Drugs were given orally through proper mixing with food so that on average, each animal would ingest 30 mg/kg DFX daily incorporated within 15 gm of dried food. For the DFX-CP group, the 15 g dried food contained CP equivalent to 30 mg/kg of animal weight. Blood samples were collected from animals using the orbital venous plexus bleeding method with the aid of ether anesthesia in special EDTA tubes on days 1, 3, and 6 after initiation of the drug treatment. Blood samples were centrifuged, and plasma was collected in Eppendorf tubes and kept at −80°C until time of analysis for DFX. On the sixth day of the treatment, animals were sacrificed by cervical dislocation with the aid of mild anesthesia. Liver, heart, kidney, spleen, and the whole gastrointestinal tract (GIT) of all animals were collected and immediately stored in 10% formalin solution for further assessment.

DFX was determined in plasma and GIT content using a slightly modified HPLC method from that employed in the solubility studies. UV detection was set at 220 nm. The mobile phase consisted of 29% acetonitrile with the same additives as mentioned earlier, and final pH was adjusted at 3.5. Plasma (250 μL) was mixed with 25 μL of mefenamic acid (80 μg/mL), which served as an internal standard, and 200 μL of acetonitrile and then centrifuged for 10 min at 7500 rpm. After that, 200 μL of the supernatant was transferred to a new tube containing 250 μL of the mobile phase. This was vortex mixed, and 100 μL of the supernatant was injected onto HPLC.

For the analysis of DFX in the small intestine, the intestinal content 60 cm next to the stomach was evacuated and vortex mixed. Then, 200 μL acetonitrile and 90 μL of tris buffer (pH 8.2) were added to about 1.5 g of the mixed content to aid dissolution of the lipophilic and acidic DFX. Samples were vortex mixed and then centrifuged for 10 min at 7500 rpm. After that, 400 μL of the supernatant solution was mixed with 500 μL of the mobile phase and 30 μL of the mefenamic acid (80 μg/mL). This was vortex mixed, and 100 μL was injected into HPLC.

**Figure 2:**
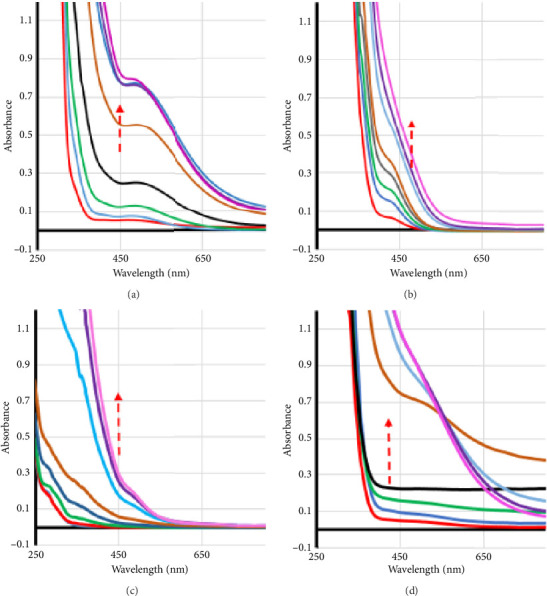
Overlaid UV spectra for the titration of (a) DFX, (b) CP, (c) blank, and (d) equimolar mixture of both DFX and CP, with iron in solutions buffered at pH 7. Similar plots obtained for the titrations at pH values of 5.2 and 2.5 are presented in Supplementary files Figures 4S and 5S, respectively.

The method was properly validated for selectivity, precision, accuracy, and linearity. Selectivity was established by demonstrating that none of the components (CP, mefenamic acid, or any of plasma components) eluted at the same retention time of DFX (Figure 2S, Supporting Information, Representative HPLC chromatograms for the determination of DFX in plasma). However, CP could not be determined by this method as it was coeluted early with other components of the plasma. Linearity was satisfactory with *R*^2^ > 0.991 in the range 1–20 μg/mL. The typical calibration equation for DFX was *y* = 0.09*x* + 0.051. For precision, five quality control samples containing both DFX and CP were prepared at low, intermediate, and high concentration levels. The obtained RSD values were in the range of 1.7%–6.8% for intermediate and high concentrations but ∼ 15% for the lowest concentration. The LOQ was determined for DFX based on a signal-to-noise ratio of 8 and was found to be 0.5 μg/mL.

For assessment of accuracy, five standard samples were prepared at low, intermediate, and high concentrations. The actual concentrations were back calculated using the obtained calibration equations. The average percentage difference between their nominal concentrations was 3.4%, and thus, the method was considered reasonably accurate. For statistical analysis, the significance of a potential difference between the average of the two groups (*N* = 8) a *t*-test was employed with the help of GraphPad online software.

### 2.8. Determination of Iron in Tissues

Accurately weighed samples of the tissues to be determined (90–120 mg for liver, and 50–80 mg for heart and spleen) were digested in 1.5 mL of concentrated H_2_SO_4_ at 60°C for two days. Samples were vortex mixed and initially diluted 12 times using 2M HCl and filtered using a 0.45 μm PTFE syringe filters. Further dilutions were made if necessary and as appropriate upon sample measurement. Samples were read on Aurora AI1200 atomic absorption spectrophotometer at the specific wavelength of iron. FeCl_3_ standard solution 1000 μg/mL (Scharlau, Spain) was used to prepare calibration curve solutions. Proper blank samples were prepared along with the calibration curve of standard iron solution. Linearity was established in the range 0.1–8 mug/mL, with a linearity coefficient > 0.99. A typical calibration equation was *A* = 0.075 + 0.02. For statistical analysis, the potential existence of a significant difference between the averages of two groups, a t-test was employed using GraphPad software.

### 2.9. Histological Assessment

Tissues of the liver, heart, kidney, and spleen, which were kept in 10% neutral buffered formalin solution, were fixed in paraffin blocks. Sections of 5 μm were taken and stained with hematoxylin and eosin before they were carefully examined under a light microscope (Optika, B-190, equipped with a camera and Liteview software, Ponteranica-Italy) at 40 and 100x magnification. Pearl's stain together with nuclear fast red as a counter stain for nuclei was used on the liver, spleen, and heart tissues to investigate the possible accumulation of iron. Slides were immersed in Pearl's stain, which was based on a 1% potassium ferrocyanide solution in 2% HCl and 10% Triton-X100, for 30 min followed by a nuclear fast red solution for 5 min.

## 3. Results

### 3.1. Characterization of the Complex

The formation of iron-DFX and iron-CP complexes was immediately evidenced by direct observation of their violet or yellow colorations, respectively. In the presence of mixtures of the two drugs together, the addition of iron resulted in a distinguished brown color suggesting the formation of a new complex most probably composed of both drugs plus iron (ternary complex; DFX-CP-Fe).

### 3.2. Absorption Spectroscopy

**Figure 3:**
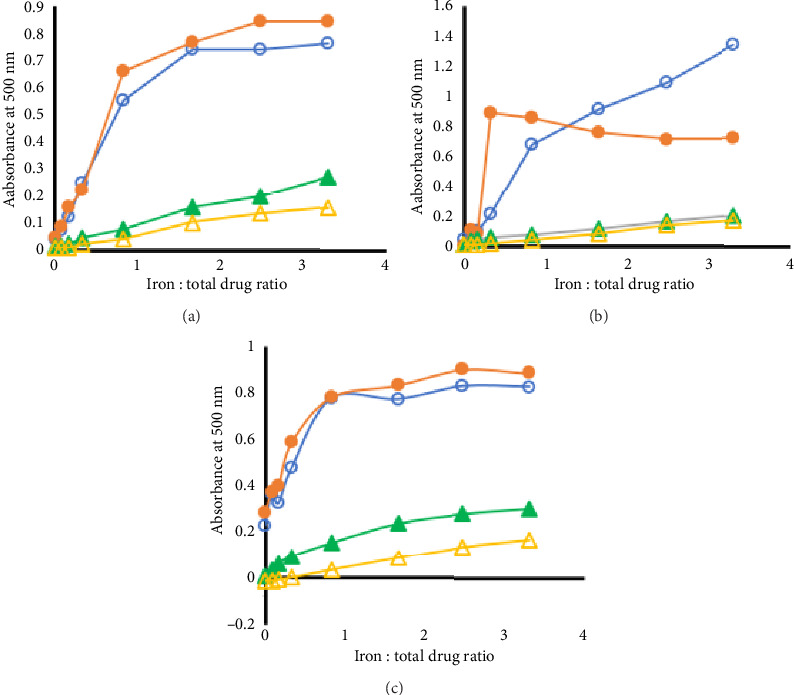
Molar ratio plots for the titration of DFX (○), CP (▲), and their equimolar mixture (•) in addition to the blank (Δ) with FeCl_3_. The experiment was performed at (a) pH 7, (b) pH 5.2, and (c) pH 2.5. In all cases, *n* = 3 and RSD < 2.2%.

A photo of the various titration sets of solutions is presented in Figure 3S, Supporting Information, Photographs for spectroscopic titrations solutions. In samples that contained both CP and DFX together, without iron, a turbidity or a precipitate was observed. Therefore, more amount of that precipitate was prepared for further characterization.

**Figure 4:**
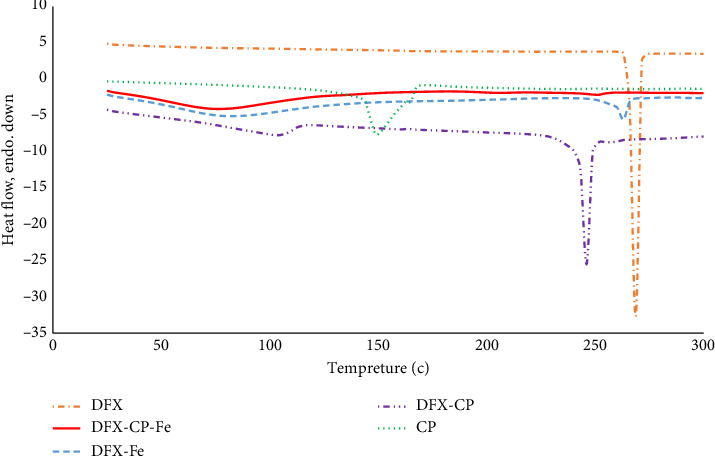
DSC thermograms for the parent compounds (DFX and CP) plus those for the different products as indicated by the key on top of the chart.

**Figure 5:**
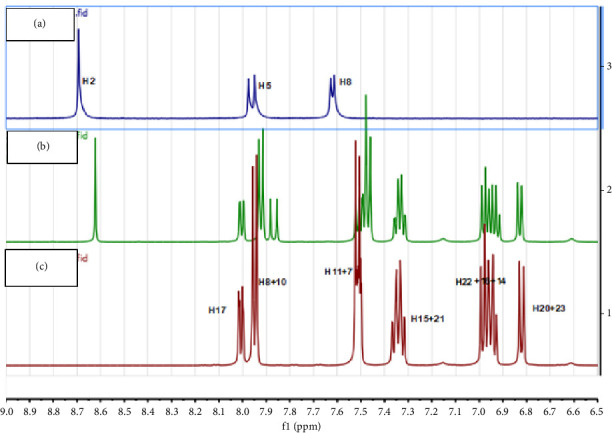
^1^H NMR spectra of (a) CP, (b) the ion pair product, and (c) DFX; proton numbering is according to Figure 1.

Absorption spectra were obtained for solutions containing fixed concentrations of DFX, buffered at pH 7 with increasing concentrations of FeCl_3_ (spectroscopic titration), showing a progressive increase in absorbance at 500 nm (Figure 2(a)). Similar titrations were made for CP alone and blank solutions (Figures 2(b) and 2(c)). The absorption spectra for solutions containing fixed equimolar amounts of both drugs, with increasing concentrations of iron, revealed a distinguished pattern (Figure 2(d)), where an isosbestic point could be observed at 548 nm for the samples with higher iron ratios. The same titration experiment was repeated with solutions buffered at pH values of 5.2 and 2.5 (Figures 4S and 5S, Supporting Information, Overlaid UV spectra for the titration of DFX at pH values of 5.2 and 2.5). At pH 5.2, the results were supportive of that at pH 7 with a clearer isosbestic point at 460 nm, observed only for the case of the mixture of the two drugs titrated together.

At pH 2.5, however, isosbestic points were observed for both cases of the DFX alone (at 630 nm) and the mixture of the two drugs (at 623 nm). Titration of CP alone did not exhibit an isosbestic point (Figure 5Sb, Supporting Information, Overlaid UV spectra for the titration of DFX at pH values of 5.2 and 2.5).

For more quantitative comparison, molar ratio plots were obtained by plotting absorbance at 500 nm against the molar ratio of added iron to the drug in each case (Figure 3). According to Figure 3(a) which summarized the findings at pH 7, the binding curve for CP was slightly different than that of the blank and thus could indicate binding to iron. However, no clear inflexion in the curve could be observed.

At pH 5.2 (Figure 3(b)), the molar ratio plot for CP alone was almost identical to that of the blank. For the cases of DFX alone or the mixtures of the two drugs, the curves plateaued at ratios iron to drug of 0.5 and 1, respectively. At pH 2.5, the two binding curves for DFX alone and the mixture were almost identical (Figure 3(c)).

### 3.3. Melting Points/DSC

The melting points were measured using the capillary method for DFX, CP, and the expected DFX: CP ion pair, and the ternary complex product. DSC thermograms were also obtained for the parent compounds and the obtained products (Figure 4). The thermogram for DFX exhibited a single sharp intense endothermic peak at 268.7°C consistent with the drug being in a crystalline form [31] and with visual observation using a capillary melting apparatus. For CP, a characteristic small and rather broad endotherm appeared at 148.7°C consistent with previous reports [32–34]. Interestingly, the product of CP and DFX (DFX-CP ion pair) exhibited a clear and significant shift in the melting endotherm of DFX (from 268.7 to 246°C), supporting ion pair formation between the positively charged CP and negatively charged DFX.

DFX-Fe complex showed a significant reduction in the endotherm of DFX with a slight shift to a lower temperature (263°C). While using the capillary apparatus, no clear melting was observed at this temperature but only shrinkage in the sample size up to 330°C. For the ternary complex (DFX-CP-Fe), there was even a larger reduction and further shift toward a lower temperature (251°C) which was seen as shrinkage in the sample size using the capillary apparatus.

### 3.4. NMR Spectroscopy

**Figure 6:**
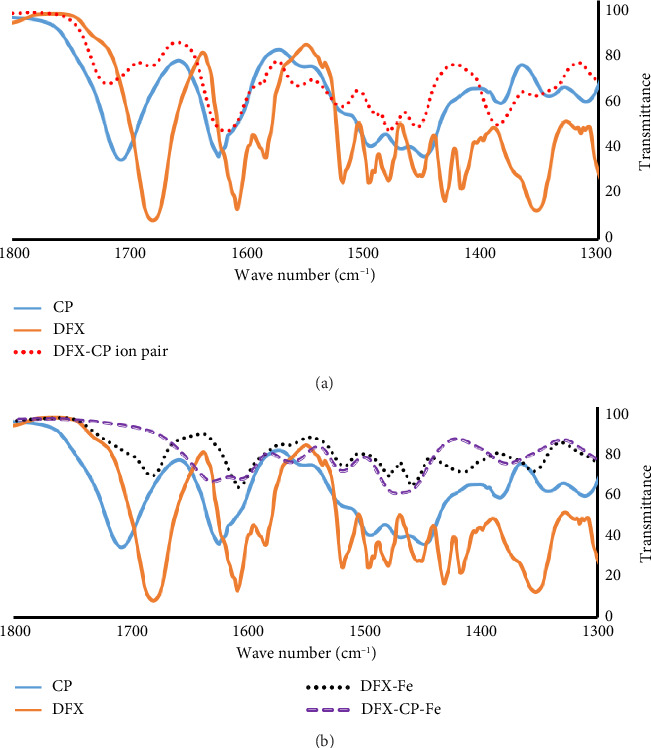
FTIR spectra for (a) the parent compounds along with the ion pair salt and (b) with the binary and ternary iron complexes.

Due to the presence of the paramagnetic iron within the complexes, their NMR spectra could not be properly investigated. However, NMR spectra were obtained for DFX, CP, and their ion pair product (DFX-CP). The aromatic region of the spectra is shown in Figure 5 while the aliphatic regions are presented in the Supporting file (Figure 6S, Supporting Information, Aliphatic region for the NMR spectra). NMR spectra for the parent compounds were in agreement with the previous reports, and peak assignments for the proton NMR spectra of DFX and CP were based on Bruin et al. [14] and Takac [35], respectively.

### 3.5. Infrared Spectroscopy

The obtained FTIR spectra for CP, DFX, and their anticipated ion pairs are shown in Figure 6(a).  Figure 6(b) shows the spectra for the iron complexes (DFX-Fe and DFX-CP-Fe) along with those for the parent compounds.

### 3.6. Percentage Content and Stoichiometric Ratio

Small samples of each CP-DFX ion pair product, DFEX-Fe, and DFX-CP-Fe were completely dissolved in a proper solvent mixture to determine the concentration of DFX in the resulting solutions by HPLC. The experimentally determined mass percentage of DFX from the taken weights corresponded to 51.5%, 87.5%, and 47% ( ± 1), respectively. Thus, the experimentally obtained percentages agreed very well with the expected theoretical percentage of DFX in the assumed products: CP-DFX ion pair (53%), (DFX)_2_Fe complex (93%), and DFX-CP-Fe complex (49%).

### 3.7. Theoretical Chemistry Calculations

Efforts were made to obtain crystals of the ternary and binary (DFX-Fe) complexes but were unsuccessful, as reported earlier by Steinhauser et al. [12]. However, crystal structures for a structurally related ligand, complexed to ferric iron, were resolved and showed two molecules of the ligand binding to one iron atom via two phenolic groups and one nitrogen of the triazole ring [12].

**Figure 7:**
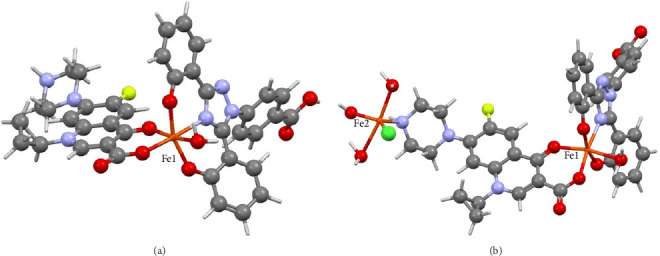
The proposed optimized structure for (a) Fe(CP) (DFX) (H_2_O) complex and (b) Fe_2_(CP) (DFX) (H_2_O)_3_OHCl. Note: green: chlorine, red: oxygen, blue: nitrogen, yellow: fluorine, and carbon: gray.

Therefore, attempts were made to obtain further support for the formation of the tertiary complexes through theoretical calculations of the thermodynamic parameters for the relevant complexation reactions. As a starting point, the binding mode of CP to the Fe^3+^ ion is selected based on its binding to other metal ions, for example, Cu(II), Co(II), Zn(II), and Cd(II) which were proven crystallographically [36]. The calculated thermodynamic parameters for a 1:1:1 ternary complex were ΔH = −66.86 kJ/mol, ΔG = 41.66 kJ/mol, ΔS = −0.0845 k J/(mol K), and Keq = 2.0 × 10^7^. A proposed optimized structure for the ternary complex in accordance with the calculated parameters is shown in Figure 7 (top).

However, UV-Vis spectroscopic titrations indicated that the ratio between the three species is DFX, CP, and iron in 1:1:2 (vide supra). The optimized structure of the iron dimer structure is shown in Figure 7 (bottom), and the thermodynamics data are ΔH° = −478.8 kJ/mol, ΔG° = −344.2 kJ/mol, ΔS° = −0.452 kJ/mol, and Keq = 2.0 × 10^60^.

### 3.8. Solubility and Apparent PCs

The obtained values of solubility for the parent compounds and the formed products are shown in Table 1. Apparent partition coefficients (PC_app_) were also obtained for the parent compounds and the formed products at pH values of 6.8 and 4.5 (Table 2). The obtained PC_app_ for DFX-CP was close to that of DFX at pH 6.8, but 1.5 times greater at pH 4.5.

### 3.9. DFX Level in Plasma and GIT Content

The HPLC method was shown to be capable of separating DFX from other plasma constituents and properly validated its quantitative determination (Figure 2S, Supporting Information, Representative HPLC chromatograms for determination of DFX in plasma). The level of DFX in plasma was determined using HPLC after 1, 3, and 6 days from the start of the treatments with either DFX alone or DFX plus CP. The obtained values are shown in Table 3. It is obvious that the levels of DFX were still low 1 day after the initiation of the treatment but seems to plateau after 3 and 6 days. After only one day of treatment, there seemed to be no significant differences between the levels of DFX among the two treatment groups. After 3 and 6 days of treatment, the group that was subjected to both drugs exhibited significantly lower concentrations of DFX, almost to one half (*p* value 0.023 and 0.011, respectively).

The level of DFX was determined in the gastrointestinal contents (within the next 60 cm of intestine beyond the stomach). The average amounts of DFX per g of contents were 17.5 and 27.5 mg for the cases of DFX alone and combined treatment groups, respectively (*p* value 0.036).

### 3.10. Iron Levels in Organs

Levels of iron in dried tissues, including blood (after extracting plasma), were determined by AAS, and the results are summarized in Table 4. With the exception of the heart, all other tissues exhibited higher concentrations of iron in both treatment groups compared to the control group. Comparing the two treatment groups together, statistically significant differences were only observed in the case of blood, where the addition of CP appeared to result in lower iron content than using DFX alone. Although this effect was more obvious at 3 days after the initiation of the treatment, the difference was still significant until the end of the treatment period (6 days).

### 3.11. Histological Assessment of Organs

**Figure 8:**
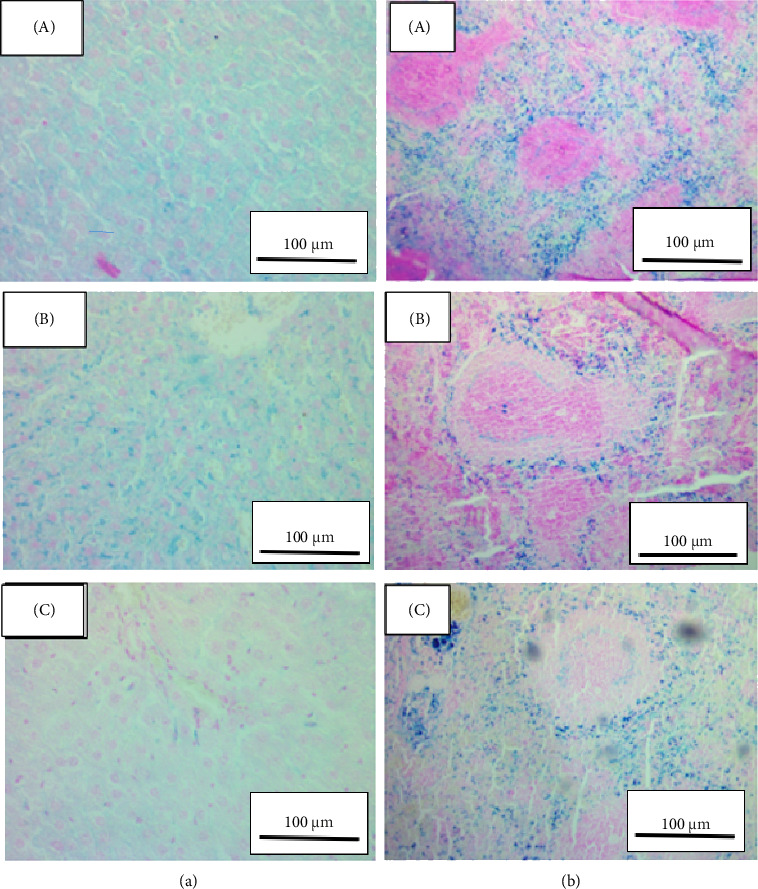
Representative photomicrographs of kidney (a) and heart (b) tissues using Pearl's stain; magnification at 100x. In both, A and B animals were subjected to high iron diet and then treated for 6 days by DFX (A) or mixture of DFX and CP (B). In (C), kidney and heart of control animals, neither subjected to iron overload nor to any other treatment.

Collected tissues of heart, spleen, kidney and liver were examined using the standard H&E stain plus the specialized Pearl's stain for detection of iron deposits. Generally, the kidney and heart tissues did not reveal significant differences between the treatment group and the control group (Figure 8).

**Figure 9:**
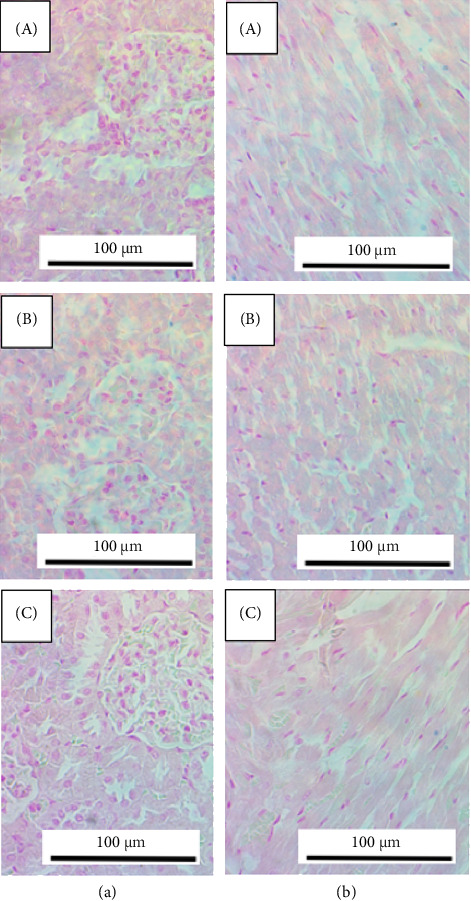
Representative photomicrographs of liver (a) and spleen (b) tissues using Pearl's stain; magnification at 100x. In both, A and B animals were subjected to high iron diet and then treated for 6 days by DFX (A) or mixture of DFX and CP (B). In (C), liver and spleen of control animals, neither subjected to iron overload nor to any other treatment.

For liver tissues, iron deposition was seen for both treatment groups contrary to the control group (Figure 9). Although the difference in the extent of iron deposited in the two treatment groups might not be so obvious, the group treated with DFX alone appeared to have a higher level of iron deposition. This effect was seen in six of eight animals. For the spleen tissue, there was a clear iron deposition, even in the control group, which had not been exposed to iron overload (Figure 9). However, the group treated with DFX alone showed clearly higher levels of deposited iron than the combined treatment group as well as the control group; the effect was seen in six out of eight animals.

## 4. Discussion

The first evidence of the involvement of CP in ternary complex formation involving DFX and iron came from the visual comparison of the pattern of color change where the presence of CP along with DFX and iron gave a distinct brown color. Initial experiments suggested the formation of an ion pair between the positively charged CP and the counter anion DFX (particularly at pH 5.2).

The presence of isosbestic points usually indicates the presence of two species, i.e., free and complexed forms. At pH 7, it was observed for the cases of DFX alone or the mixture of both drugs together but not for the titration of CP alone or for the blank. Moreover, the observed distinguished spectral changes in Figure 2(d) could not be merely explained as the arithmetic sum of the responses recorded for the individual components. For example, the sum of the absorbance values of all individual components at 650 nm could not mount to 0.3 (Figures 2(a), 2(b), 2(c)), but the recorded value for the titration of the mixture was > 0.45, i.e., 50% higher than the theoretically expected value if it was simply the sum of the absorbance contributions of the individual complexes. Thus, a ternary complex that involved CP and DFX (DFX-CP-Fe) was most likely formed.

A similar argument can be made at pH 5.2. Therefore, the formation of the ternary complex at pH 5.2 is supported. However, for pH 2.5, all spectra recorded for the mixture could fit the sum of the absorbance values recorded for the individual components at a particular ratio and wavelength. Therefore, the data at pH 2.5 did not support the formation of the ternary complex but only of the binary one. It could be concluded that only DFX contributed to complex formation at the low pH of 2.5. That was consistent with the fact that the carboxyl group of CP is important to its ability to form chelate metal complexes, where at very low pH values, the carboxyl group would be largely protonated and thus with a reduced ability to form complexes. On the other hand, DFX is known to form very stable iron complexes through the phenolic hydroxyl groups.

According to Figure 3(a), it could be concluded that DFX forms a 1:1 complex with iron when alone but a ternary complex comprising DFX, CP, and iron in 1:1:2 ratio when existing with CP at pH 7. The support to that conclusion came from the observation that the curves plateaued at a nearly 1:1 ratio of iron to the total drug in both cases. At pH 5.2 (Figure 3(b)), while there was no evidence of complexation of CP to ferric iron, the evidence for the formation of a ternary complex was quite obvious. At pH 2.5, the presence of CP did not alter the complexation between DFX and iron, i.e., only a binary 1:1 DFX:iron complex was formed. The observation that 1:1 (DFX:Fe) is favored at a low pH is consistent with previous reports [11]. Thus, the molar ratio plots supported the above discussion and conclusion pertaining the overall spectral shapes and the isosbestic point for DFX at pH 2.5.

DSC provided evidence on the formation of a CP-DFX ion pair. CP was previously shown to form ion pair salts with various organic anions [32], but this is the first report of potential ion pair formation between CP and DFX. The formed DFX-CP ion pair is expected to have different lipophilicity than the parent compound. Therefore, concomitant use of CP is likely to modify the therapeutic action of DFX irrespective of the chelate complex formation mechanism, e.g., through influencing absorption or excretion. DSC also provided evidence of the formation of ternary complex, CP-DFX-Fe, because the presumed product exhibited a different thermogram than other tested materials.

In the NMR experiment, all three aromatic protons of CP were obviously shifted; only protons number 11 + 7 and 8 + 10 of DFX showed significant shifts toward lower frequencies (Figures 5 and 6S). This observation was consistent with the binding of the two drug molecules taking place through the electrostatic attraction between the negatively charged carboxyl of DFX and the positively charged amino group of CP. Thus, the aromatic protons of the benzoic acid moiety in DFX, being closest to the negative charge, are expected to be the highest shifted on the NMR spectra. This is also supported by the observation that the aliphatic protons within the piperazine ring of CP were highly downfield shifted in accordance with being closer to the center of the positive charge, thus constituting additional evidence of DFX-CP ion pair formation.

The obtained FTIR spectra for the parent compounds generally agreed with the previously reported spectra. Focusing on the carbonyl absorption band, as a clear representative, it appeared at 1709 and 1680 cm^−1^ for CP and DFX, respectively [31, 37]. In the spectrum for the ion pair (DFX and CP), they were shifted to a longer frequency and appeared at 1719 and 1682 cm^−1^, respectively, together with the band for DFX becoming less intense than that of CP. These changes support the formation of an ion pair salt between DFX and CP and not simply having a physical mixture of two independent compounds.

The carbonyl absorption band did not appear to change significantly when DFX bound to Fe^3+^. However, in the ternary complex, the two carbonyl bands (from CP and DFX) seemed to coalesce and were significantly shifted to lower frequency (1632 cm^−1^). These observations are in accordance with the formation of stronger coordinate bonds involving the carboxyl group leading to the weakening of the carbonyl group, and thus, its absorption band shifted to a lower frequency. Overall, FTIR spectra provided clear evidence of the formation of a ternary complex, involving iron, DFX, and CP, with different spectral features than the binary DFX-Fe complex.

When the percentage of DFX was calculated from the HPLC analysis, the percentage content of DFX supported the formation of the anticipated complexes. The obtained ratio of iron in the ternary complex differed from that shown by spectroscopic titration. However, the sample analyzed by HPLC was precipitated in the solid state out of a hydromethanolic solution with pH adjusted at 8.2, which might explain the differences in the obtained ratio of iron within the complexes.

The formation of a ternary complex with the formula [Fe(CP) (DFX) (H_2_O)] was also shown to be thermodynamically favored at 25°C as indicated by the calculated thermodynamics parameters. The more negative value of the free energy for the iron dimer structure and larger Keq value indicated that the dimeric structure is thermodynamically favored over the monomeric iron structure in an aqueous system, which agrees with the UV-Vis data. The reaction of the formation of the dimeric structure from its constituents goes to completion due to the extremely high absolute value of the free energy. Therefore, this ternary complex form is quite likely to be maintained within biological systems. It is noteworthy to mention that the chloride anion was used to balance the charge. The geometry around the iron ion in the monomeric species is distorted octahedral, whereas there are two different geometries around the iron ions in the dimeric species (distorted octahedral (Fe1; Figure 7) and distorted and trigonal bipyramidal (Fe2; Figure 7)).

The reported water solubility for DFX varied between 4 and 38 μg/mL [38, 39]. However, the value obtained under our experimental conditions, at nearly neutral pH (6.8), was 140 μg/mL, which might be due to the fact that DFX is a carboxylic acid and expected to exist predominantly in its ionized form at that pH. Therefore, DFX exhibited a pH-dependent solubility profile in the studied range. The ion pair salt of DFX-CP had lower solubility values than native DFX at all pH values examined although to various extents. With this obvious effect on solubility, it could be concluded that the association between DFX and CP is strong enough to be maintained in their diluted solutions.

For the complex DFX-Fe, the measured solubility was less than half the value for parent DFX at both pH values of 6.8 and 4.5. At pH 1.2, although the solubility of the iron complexed DFX was less than its free form, it did not mount to its half. This was consistent with the expectations that the complexation efficiency decreases with decreasing pH. For the ternary complex, its solubility was about 16% less than that of free DFX at pH 6.8 with lower differences in solubilities at lower pH values.

The PC_app_ values for DFX-Fe were 3 and 5 times lower than those for DFX at pH values of 6.8 and 4.5, respectively. Most importantly, perhaps, the values for DFX-CP-Fe were >200 times and 4 times lower than those for free DFX at pH values of 6.8 and 4.5, respectively. This was particularly interesting because the corresponding differences in their aqueous solubility values did not parallel those great differences in PC_app_. Overall, these differences in solubility values and PC_app_ might be sufficient to influence the pharmacokinetics, distribution, and pharmacodynamics of DFX as a result of the ternary complex formation with CP and Fe^3+^.

Measurements of DFX in rat plasma showed a decrease in its level when coadministered with CP. It is difficult to explain these findings in terms of the effect of CP on the metabolizing enzymes because CP was previously shown to be an inhibitor rather than an inducer of the enzymes [40–42]. Another possible mechanism that could explain the observed interaction was through interference with the absorption or the excretion processes of DFX in the GIT. That could be simply a result of the differences in physicochemical properties of the complex in comparison to the free drug or due to interference with the cellular transport systems for DFX.

When DFX was determined in the proximal GIT contents of the animals, the group that received DFX alone showed a significantly lower level although an opposite picture was seen in plasma. These findings suggest that the interaction between CP and DFX was due to either a decrease in absorption or an increase in hepatic excretion of DFX, perhaps in its iron complexed form, in the intestine. Interference of CP or the anticipated ternary complex with the intestinal organic anion transporter system is also other possibilities to account for the observed effect. In all cases, the above discussed solubility and lipophilicity differences between binary and ternary iron complexes (DFX-Fe versus DFX-CP-Fe) are quite likely the reason behind the obtained experimental data.

Iron levels in the blood of the group subjected to combined treatments was statistically lower, i.e., a more pharmacodynamic effect. In tissues, chelation therapy was more pronounced on cardiac tissue than on the liver and spleen in both groups. Direct comparison of the effects of the two treatments on cardiac tissue revealed no statistically significant differences. However, the combined treatment group exhibited a significantly lower (*p* = 0.034) iron content compared to the control group. Therefore, this could be indirect evidence that the addition of CP to DFX may result in the potentiation of the chelation effect of DFX. Achieving lower iron levels in the blood and heart and at the same time lower plasma levels of DFX, as a consequence of coadministration of CP, could only be explained by the differences in physicochemical properties of the ternary complex leading to its more efficient excretion.

Attempts were made to investigate different potential effects of the two treatments on various organ weights. No significant differences could be detected between the weights of any organs in the two groups. However, taking the ratio of the weight of heart to that of the spleen, a statistically significant difference between the two groups was established (*p* = 0.027). The calculated ratios were 0.83 and 1.06 for the groups treated with DFX alone and DFX plus CP, respectively. Finally, these findings showed clearly that the presence of CP along with DFX resulted in modulating the pharmacodynamic effects of the latter.

Histological assessment of the spleen, in particular, also showed a higher deposition of iron in the DFX treatment group in comparison to the combined treatment group. Overall, in this pilot study, evidence suggests that combined treatment with both DFX and CP may result in a more pronounced effect on iron clearance and at the same time lower levels of DFX in plasma. In actual clinical use, if these findings were ascertained, then the concomitant use of CP with DFX might be advantageous to minimize the potentially serious side effects of the latter while enhancing or at least maintaining the pharmacodynamic effect of DFX [43].

## 5. Conclusion

The formation of a ternary complex involving DFX, CP, and ferric iron *in vivo* is quite possible. That conclusion was supported by spectroscopic techniques, HPLC, and DSC thermograms in addition to theoretical chemistry calculations. Theoretical chemistry calculations showed that the formation of two ternary complexes, with different ratios of iron (monomeric and dimeric iron), was favorable with estimated thermodynamic equilibrium constants of 2.0 × 10^7^ (for monomeric iron) and 2.0 × 10^60^ (for dimeric iron). The very high constant of the dimeric form ensures maintaining the complex intact in the body.

The ternary complex was prepared, obtained in the solid state (monomeric iron), and characterized. The ternary complex exhibited lower solubility and lower PC values than the binary complex of DFX and iron. The plasma level of DFX was found to decrease to almost half upon coadministration of CP. Coadministration of CP with DFX resulted in lower levels of iron in blood. Using AAS, the level of deposited iron was not statistically significant in other organs including the spleen and liver. However, using Pearl's staining, the coadministration of CP resulted in less deposition of iron in the spleen and, to a much lower extent, in the liver. Overall, this is the first report on the potential interaction between DFX and CP, and further work is needed to better characterize the influence of CP in actual clinical settings. Assuming that the results of this work were further confirmed, the coadministration of CP with DFX might offer an advantage of achieving the desired iron lowering effect but at lower concentration of the potentially toxic DFX.

## Figures and Tables

**Table 1:** Measured saturation solubility of the different compounds at different pH values examined.

Compound	Solubility in μg/mL (±SD)		
	pH 6.8	pH 4.5	pH 1.2
DFX	140.8 (1.9)	19.1 (5.4)	9.9 (8.3)
DFX-CP	86.2 (1.4)	22.9 (5.3)	4.5 (18.2)
DFX-Fe	61.7 (2.7)	7.0 (9.8)	5.2 (18.4)
DFX-CP-Fe	51.6 (3.5)	8.7 (9.5)	3.7 (17.4)

*Note:* Standard deviations (SDs) are shown between brackets (*n* = 3).

**Table 2:** Apparent partition coefficients of the different compounds measured at pH 6.8 and 4.5.

	Apparent partition coefficient (±SD)	
	pH 6.8	pH 4.5
DFX	7.9 (1.11)	2.1 (5.7)
DFX-CP	8.8 (2.7)	3.2 (3.3)
DFX-Fe	2.4 (1.1)	0.4 (3.1)
DFX-CP-Fe	0.01 (1.9)	0.52 (3.8)

*Note:* Standard deviations (SDs) are shown between brackets (*n* = 3).

**Table 3:** Level of DFX in plasma of the two groups of animals at three time intervals since the start of addition of the drugs to their food.

Time of sampling	Level of DFX in plasma in μg/mL (SD)		*p* value at 0.05 significance level
	Group treated with DFX alone	Group treated with DFX + CP	
1 day	1.2 (0.4)	0.13 (0.4)	0.346
3 days	10.7 (7.1)	5.2 (2.4)	0.023
6 days	9.7 (4.0)	4.8 (3.9)	0.011

*Note: N* = 8 in each group, the values shown in brackets are the standard deviations.

**Table 4:** Level of iron in blood and other tissues determined by AAS.

	Experimentally found content of iron in dried tissue in mg/100g of tissue (SD)			*p* value		
	DFX	DFX + CP	Control	DFX vs control	DFX + CP vs control	DFX vs DFX + CP
Dried blood (3d)∗	4.73 (0.2)	3.7 (0.2)	3.4 (0.16)	0.00074	0.2078	0.012
Dried blood (6d)∗	4.94 (0.2)	4.40 (0.1)	3.4 (0.16)	0.00005	0.00007	0.022
Liver	162.6 (10.1)	158.6 (5.9)	111.1 (8.0)	0.00018	0.0013	0.367
Spleen	168.8 (9.8)	172.0 (6.3)	92.5 (9.1)	0.00007	0.00001	0.394
Heart	50.3 (2.7)	43.7 (5.1)	61 (7.6)	0.074	0.03364	0.136

Note: *n* = 8. Standard deviation (SD) error of the mean is shown in bold between brackets.

^∗^3d and 6d refer to three and 6 days after initiation of iron chelation therapy, respectively.

## Data Availability

Extra data are available upon a reasonable request.

## Supporting Information

Figure 1S: Representative HPLC chromatograms for the determination of DFX in solubility studies. Figure 2S: Representative HPLC chromatograms for determination of DFX in plasma. Figure 3S: Photographs for spectroscopic titrations solutions. Figure 4S: Overlaid UV spectra for the titration solutions at pH of 5.2. Figure 5S: Overlaid UV spectra for the titration solutions at pH of 2.5. Figure 6S: Aliphatic region for the NMR spectra of CP, DFX, and their complex.
