# Supplementary material for: Deferasirox and Ciprofloxacin: Potential Ternary Complex Formation With Ferric Iron, Pharmacodynamic, and Pharmacokinetic Interactions
Source: ScientificWorldJournal. 2024 Nov 29;2024:9309491. doi: 10.1155/tswj/9309491 (PMC11623990; doi:10.1155/tswj/9309491)
Supplement: Supporting Information — Additional supporting information can be found online in the Supporting Information section. [file 9309491.f1.docx]

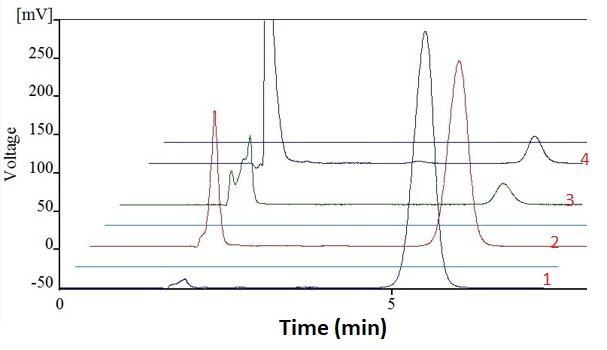


Fig. 1S: Representative HPLC chromatograms for the determination of DFX in solubility studies. Peak identification: DFX at about 5 min and CP at about 2 min. In 1, DFX alone, in 2 a standard mixture of DFX and CP, in 3 a sample of DFX-CP-Fe complex and in 4 a solubility sample for the product DFX-CP at pH value of 6.8.


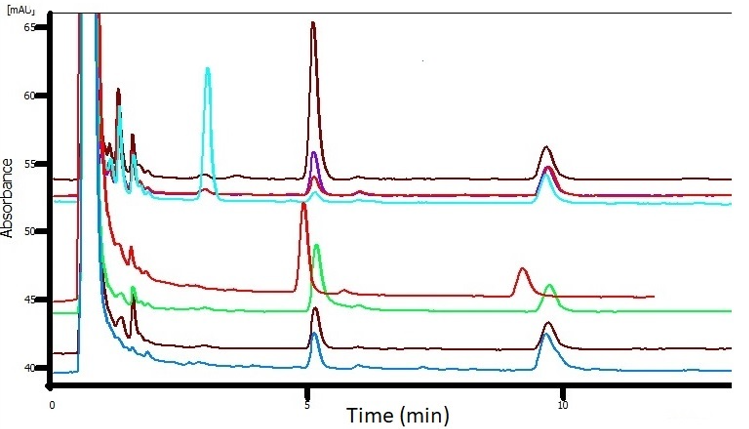


Fig. 2S: Representative HPLC chromatograms for determination of DFX in plasma. Peak identification: DFX at about 5 min and mefenamic acid (internal standard) at about 9.5 min. The four uppermost chromatograms belong to a set of calibration curve solutions at 0.5, 1, 2 and 14 mg/ml level of DFX. The two chromatograms in the middle correspond to two rat plasma samples for the group taking DFX alone. The two chromatograms at the bottom belong to two rat plasma samples in the group treated with both of DFX and CP.


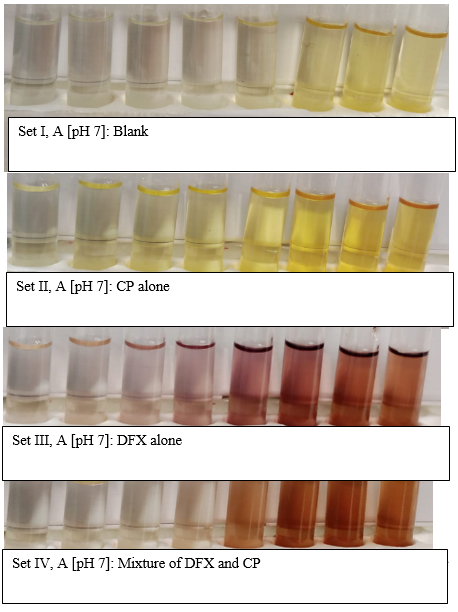


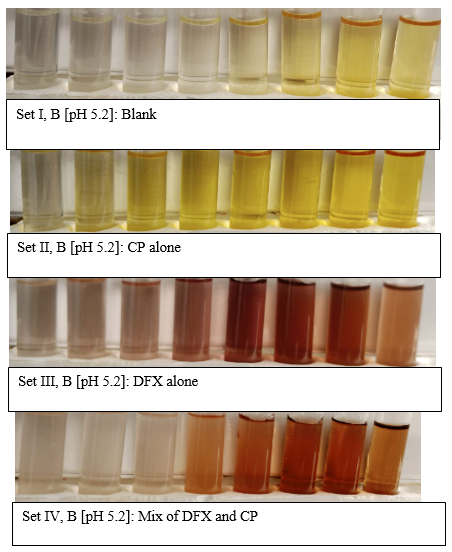


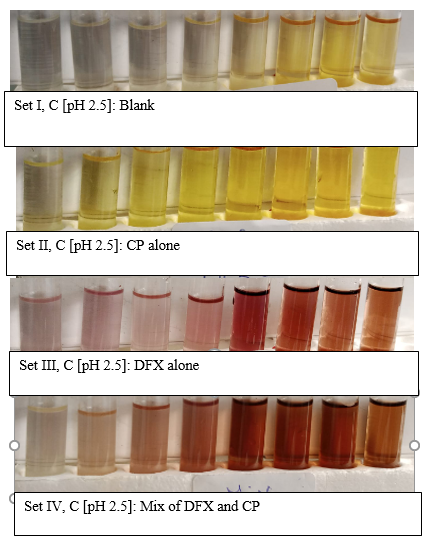


Fig. 3S: Photographs for spectroscopic titrations solutions of CP alone (set II), DFX alone (set III), mixture of the two drugs (set IV) and the blank (set I). Groups A, B and C represent pH values of 7, 5.2 and 2.5, respectively.


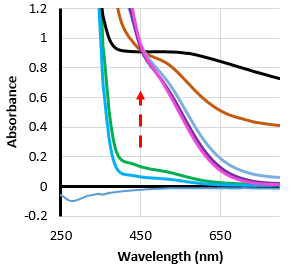

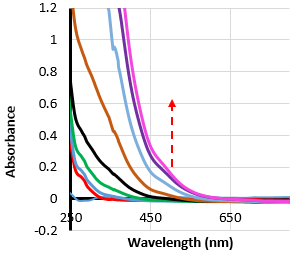

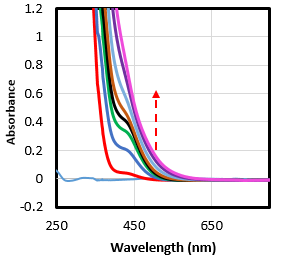

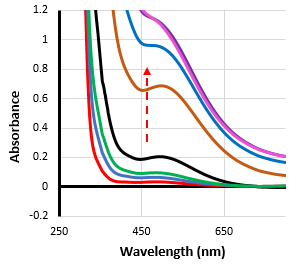


**D**

**C**

**B**

**A**

Figure S4: Overlaid UV spectra for the titration of DFX (A), CP (B), blank (C) and equimolar

mixture of both DFX and CP (D) with iron in solutions buffered at pH of 5.2.


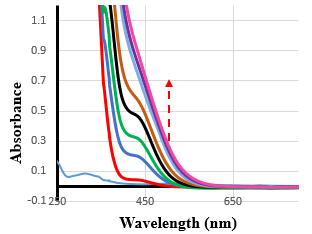


**B**

**A**


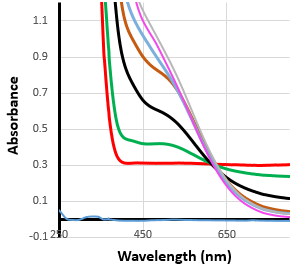

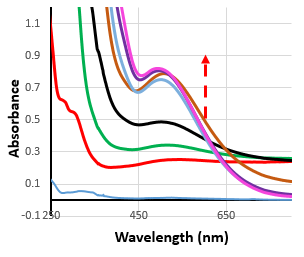


**D**

**C**


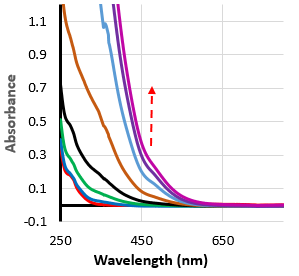


Figure S5: Overlaid UV spectra for the titration of DFX (A), CP (B), blank (C) and equimolar

mixture of both DFX and CP (D) with iron in solutions buffered at pH of 2.5.


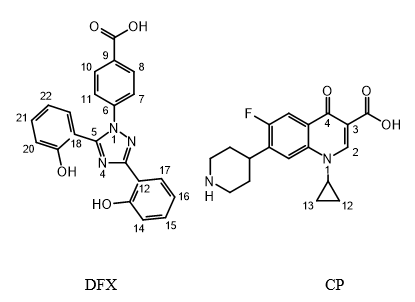


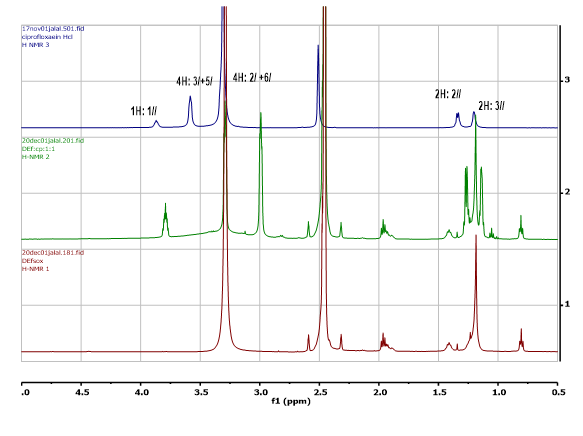


**C**

**B**

**A**

Fig. 6S: Aliphatic region for the NMR spectra of CP (a), DFX-CP (b) salt and DFX (c).
